# Adaptive monitoring of coral health at Scott Reef where data exhibit nonlinear and disturbed trends over time

**DOI:** 10.1002/ece3.9233

**Published:** 2022-09-11

**Authors:** Pubudu Thilan Abeysiri Wickrama Liyanaarachchige, Rebecca Fisher, Helen Thompson, Patricia Menendez, James Gilmour, James M. McGree

**Affiliations:** ^1^ School of Mathematical Sciences, Faculty of Science Queensland University of Technology (QUT) Brisbane Queensland Australia; ^2^ Australian Research Council Centre of Excellence for Mathematical and Statistical Frontiers (ACEMS) Brisbane Queensland Australia; ^3^ Centre for Data Science, Queensland University of Technology Brisbane Queensland Australia; ^4^ Department of Mathematics University of Ruhuna Matara Sri Lanka; ^5^ Australian Institute of Marine Science Crawley Western Australia Australia; ^6^ Oceans Institute University of Western Australia Crawley Western Australia Australia; ^7^ Department of Econometric and Business Statistics Monash University Clayton Victoria Australia; ^8^ Australian Institute of Marine Science Townsville Queensland Australia

**Keywords:** ecological monitoring, interrupted time series regression, mass bleaching events, semiparametric regression, sudden declines in trends

## Abstract

Time series data are often observed in ecological monitoring. Frequently, such data exhibit nonlinear trends over time potentially due to complex relationships between observed and auxiliary variables, and there may also be sudden declines over time due to major disturbances. This poses substantial challenges for modeling such data and also for adaptive monitoring. To address this, we propose methods for finding adaptive designs for monitoring in such settings. This work is motivated by a monitoring program that has been established at Scott Reef; a coral reef off the Western coast of Australia. Data collected for monitoring the health of Scott Reef are considered, and semiparametric and interrupted time series modeling approaches are adopted to describe how these data vary over time. New methods are then proposed that enable adaptive monitoring designs to be found based on such modeling approaches. These methods are then applied to find future monitoring designs at Scott Reef where it was found that future information gain is expected to be similar across a variety of different sites, suggesting that no particular location needs to be prioritized at Scott Reef for the next monitoring phase. In addition, it was found that omitting some sampling sites/reef locations was possible without substantial loss in expected information gain, depending upon the disturbances that were observed. The resulting adaptive designs are used to form recommendations for future monitoring in this region, and for reefs where changes in the current monitoring practices are being sought. As the methods used and developed throughout this study are generic in nature, this research has the potential to improve ecological monitoring more broadly where complex data are being collected over time.

## Graphical abstract

When complex time series data are observed, new methods for adaptive monitoring are proposed to make recommendations for future coral reef monitoring in the Western coast of Australian, as well as for reefs where changes in the current monitoring practices are being sought.

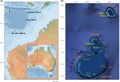

## 1 INTRODUCTION

Coral reefs are one of the most beautiful and biologically diverse ecosystems globally. Unfortunately, environmental stressors such as severe cyclones and bleaching events have had a negative impact on coral reefs (Gilmour et al., 2019). As a result, the health of coral reefs is continually being monitored to estimate the impact of such disturbances and to identify additional vulnerabilities to decline.

In long‐term coral reef monitoring, experimental design plays a vital role in creating survey designs to collect data for assessing coral health, trends over space and time, and to identify vulnerabilities of coral communities to different disturbances (Campbell et al., 2001). Broadly, there are two types of designs: static and adaptive. Static designs are those that do not change over time (e.g., the same sites/reefs are visited each year) and have been commonly used within monitoring programs. In contrast, adaptive designs can vary over time based on, for example, information from new data, and such methods have been proposed recently for determining when and where to sample within a coral reef to monitor coral health (Kang et al., 2016).

In the context of adaptive design, the adaptation can be informed by a statistical model. The purpose of this model is to extract information contained within the historical data to quantify uncertainties about, for example, the model itself, the model parameter values, and the response variable of interest, and then utilize this information to guide future surveys. For example, in Thilan et al. (2021), a spatial Beta regression model was developed for hard coral cover, and used to find future adaptive designs. When such designs were compared with those based on a linear model, the importance of appropriately capturing trends and variability within the data was highlighted as this led to more informative and therefore more efficient designs.

Ecosystems are subjected to a variety of observed and unobserved impacts which may interact in a variety of different ways (Newbold et al., 2020). For instance, coral reef ecosystems often exhibit nonlinear trends including sudden shifts due to mass coral bleaching, severe storms, and crown‐of‐thorns starfish (COTS) outbreaks (Done, 1992; McCook, 1999). These nonlinear trends pose significant challenges in modeling ecological data (Oddi et al., 2019), and this challenge is further exacerbated when there are sudden shifts in the overall trend due to major disturbances (Scheffer et al., 2001).

Generally, semiparametric regression modeling approaches provide more flexibility than parametric models in describing a variety of relationships between (a function of) the mean response and given covariates (Crainiceanu et al., 2005). Thus, the development and use of semiparametric regression modeling approaches has received attention recently for modeling ecological data (Vercelloni et al., 2014, 2017). However, little guidance is available for finding adaptive designs based on such models which limits how such information can be used to guide future reef monitoring. In addition, to account for sudden or sharp declines in the mean response due to disturbances such as a mass bleaching event, approaches from time series regression modeling can be considered. Within a monitoring program, of further interest is then how the coral reef should be sampled to estimate the impact of such a disturbance.

In this paper, we propose new methods to find adaptive designs when the historical data exhibit nonlinear trends and sudden declines over time. The motivation for this research is the improvement of the Scott Reef Research Program (SRRP); a monitoring program of a coral reef system off the Western coast of Australia. We leverage information from the historical data through semiparametric and time series modeling approaches. Methods for finding adaptive designs based on such a modeling approach are then proposed, and designs are found under future monitoring scenarios at Scott Reef. These designs are then evaluated and used to provide recommendations for future surveys at Scott Reef and other reef monitoring programs where changes in the sampling practices are being contemplated.

## 2 MOTIVATING EXAMPLE

**FIGURE 1:**
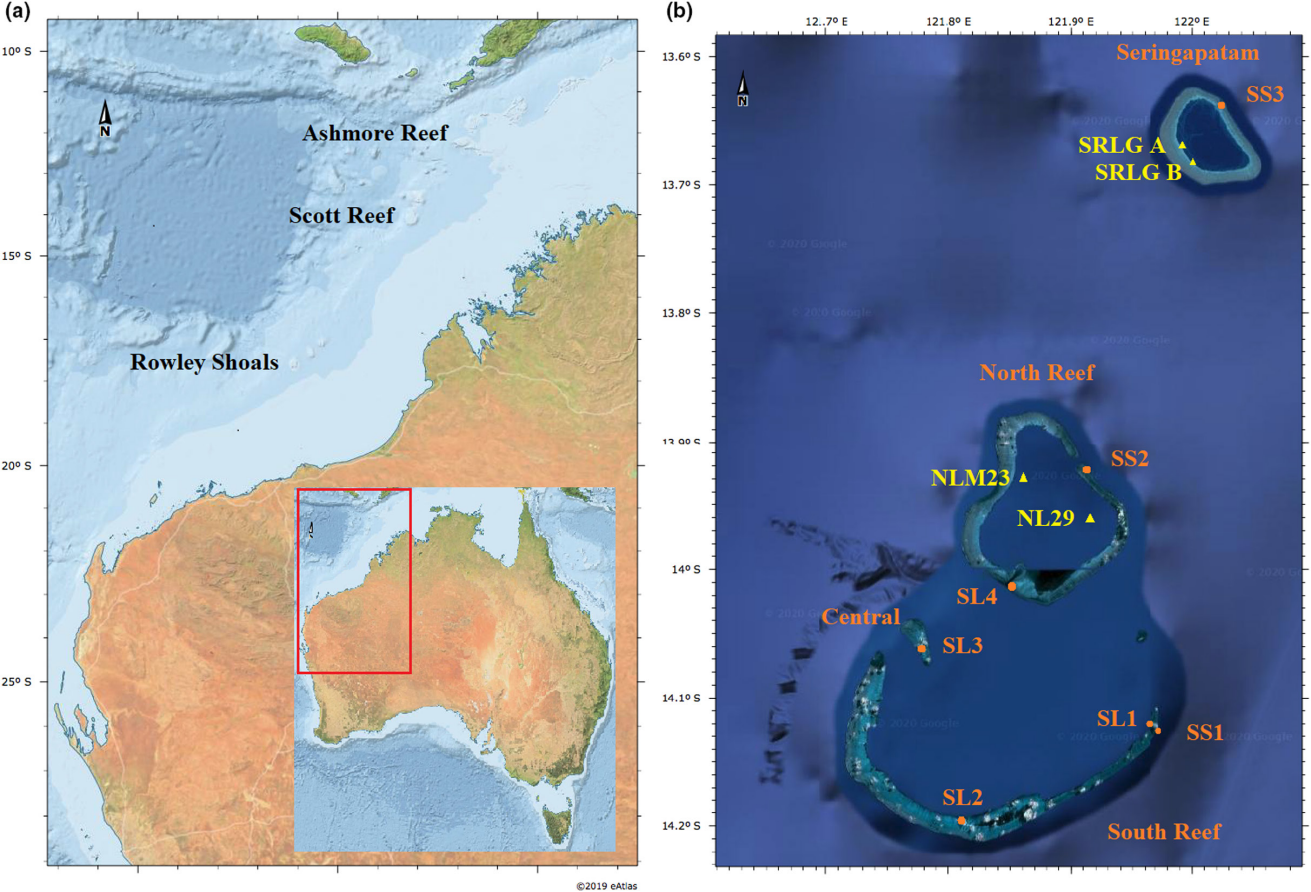
(a) The location of the system of Scott reef and (b) the long‐term monitoring sites located at south reef, central, north reef, and Seringapatam (Google maps, n.d.). The orange points represent sites that have been surveyed since 1994 and the yellow triangles represent newly added sites after the 2016 bleaching event (Sourced from: Bright Earth eAtlas basemap v1.0, AIMS).

Scott Reef is located 270 km off the coast of North‐Western Australia (Gilmour & Smith, 2013; Figure 1a) and accordingly is isolated from many human impacts. However, these reefs are frequently exposed to cyclones and bleaching events. For example, due to elevated water temperatures over a few months in 1998, Scott Reef experienced a mass bleaching event, resulting in a decline of coral up to 80% (Gilmour et al., 2019; Gilmour & Smith, 2013) and thus, a complete change in hard coral cover trends was observed over time (Figure S1). Furthermore, such disturbance exposure did not seem homogeneous across different survey locations. That is, there were survivors or relatively unharmed, moderately, and severely affected reef locations after this severe disturbance event (Figure S2; Gilmour & Smith, 2013). By adequately identifying the impacts of sudden disturbances, variations across the reef, and potential causes, it should be possible to develop efficient and appropriate monitoring practices that can change/evolve over time, and this is the aim of this paper.

## 3 DATA

The system of Scott Reef comprises of four separate structures, namely North Reef, Central, South Reef, and Seringapatam (Figure 1b). Under the SRRP, data have been collected over six habitats called slope, upper slope, crest, flat, lagoon, and outcrop from 1994.83 to 2017.92, where decimals represent survey times within a given year, that is, 0.83 denotes the 10th of 12 months. Three core sites have been sampled to collect data, which are nested within each of seven reef locations (i.e., SL1, SL2, SL3, SL4, SS1, SS2, and SS3; Figure 1b). As part of the SRRP surveys, the relative abundance of different coral types (e.g., hard coral and soft coral) is recorded at each site. In this study, we consider hard coral cover as an indicator of coral health (Bruno & Selig, 2007; Osborne et al., 2011). In Scott Reef, hard coral cover proportions are evaluated based on five points being randomly placed on each image, and the proportion of points that are placed on coral determines the measured coral cover. Given there are 50 images for each of five transects, there are 1250 data points per site that may have been randomly placed on coral. In this study, data collected between 1994.83 and 2016.08 were considered to develop future monitoring plans at Scott Reef (Section 4).

Scott Reef research program surveys are typically conducted in October, but variations have been observed from year to year (Table S1). For instance, when there was a severe disturbance, Australian Institute of Marine Science (AIMS) has collected data during, immediately after, and then later in the year depending upon the nature of the disturbance. In 1998, they conducted such pre‐ and post‐bleaching surveys in January and October, respectively, where there was interest in quantifying coral loss during this time.

The record of disturbance data refers to what occurred between sampling times. Accordingly, bleaching exposure has been recorded as either present or not (i.e., 0 = No coral bleaching, 1 indicating ≥1% coral bleached) for the whole reef system. Similarly, cyclone exposure has been recorded in terms of the number of hours the reef system was exposed to damaging waves (Puotinen et al., 2016). These covariates vary over time for the whole reef system, and thus are hereafter referred to as time‐varying covariates. In addition, reef location‐specific (rather than whole of reef) disturbance data for bleaching and cyclone exposure have also been recorded.

## 4 MONITORING OBJECTIVES

This study aims to develop recommendations for future monitoring at Scott Reef and other reef monitoring programs where changes are being considered. We aim to achieve this goal through considering the following two questions which form the basis for our two objectives:

(1) Are some reef locations (i.e., SL1, …, SS3) within Scott Reef more important than others in providing information on hard coral cover?

(2) Which site at each reef location provides the most information about hard coral cover?

These two objectives will be addressed through the use of an adaptive design approach which is described in the next section.

## 5 DESIGN FRAMEWORK

**FIGURE 2:**
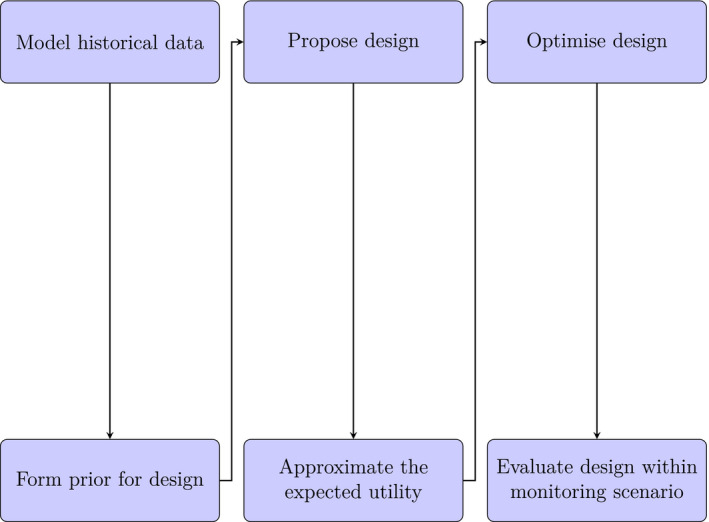
Diagram of the proposed Bayesian adaptive design framework. This consists of three stages: quantifying prior information (left), assessing designs (middle), and optimization and evaluation (right).

Throughout this paper, we consider a Bayesian design framework as information from historical data can be leveraged to inform design selection. There are also other benefits of such a framework including flexible choice of utility functions (e.g., monitoring objectives) and rigorous handling of uncertainty. The approach to find a design in this framework can be split into three stages, as shown in Figure 2. The first stage entails quantifying prior information about the ecological process being monitored, and this is achieved here by modeling historical data collected on Scott Reef. Through building a Bayesian statistical model for these data, we will form a posterior distribution of the parameters. It is this distribution that will be used as the prior information for design selection. In the second stage, this prior information is exploited to assess the value of different designs in addressing proposed monitoring objectives (defined above). To do so, an expected utility function (Chaloner & Verdinelli, 1995) is used which evaluates the information that is expected to be gained from running a given design. Then, given the value of a design can be quantified, the last stage of the process is to optimize the choice of design with respect to the monitoring objective for a given future monitoring scenario. Once such a design has been found, it is proposed as the optimal design, that is, the design that is expected to provide the most information about a given monitoring objective within the monitoring scenario. In the next section, we describe each of these three stages in more detail.

### 5.1 Quantifying prior information

#### 5.1.1 Model historical data

Semiparametric regression approaches can be used to capture nonlinear relationships within a regression model and have been considered previously to describe data from coral communities on the Great Barrier Reef (GBR; Vercelloni et al., 2014). Here, we develop a model to capture nonlinear trends in hard coral cover $y_{\mathrm{srt}}\;$ at the s‐th site, in the r‐th reef location at the t‐th survey time $\left(s=1,2,3;r=1,\ldots ,7;t=1994.83,\ldots ,2016.08\right)$. We assume that $y_{\mathrm{srt}}$ follows a Binomial distribution (denoted as “BIN”) such that $y_{\mathrm{srt}}∼\mathrm{BIN}\left(n,\pi_{\mathrm{srt}}\right)$ where the first parameter n is number of trials and the second parameter $\pi_{\mathrm{srt}}$ is the probability of success. Here, n is the number of points in a 250 m combined transect length, that is, n=1250, and $\pi_{\mathrm{srt}}$ can be expressed as $\pi_{\mathrm{srt}}=1/\left(1+\exp\left(-\mu_{\mathrm{srt}}\right)\right)$ where $\mu_{\mathrm{srt}}$ denotes the linear predictor of the model, that is, a linear combination of parameters and covariates. Within this linear predictor, a semiparametric modeling approach can be used to provide flexibility in describing the relationship between covariates and the mean response $\mu_{\mathrm{srt}}$. This is achieved through the inclusion of an additive term $f\left(x_{\mathrm{srt}}\right)$ where $f\left(\cdot \right)$ is some smooth function of covariate $x_{\mathrm{srt}}$ which could be (for example) the sampling year.

There are different methods for modeling the smooth function, including cubic splines, B‐splines, truncated polynomials, and radial splines (Crainiceanu et al., 2005). We consider the low‐rank thin‐plate splines approach as it requires fewer parameters to estimate, and also it is relatively insensitive to the choice of knots (Wood, 2003). Such a smooth function can be expressed as follows:

(1) $$f\left(x_{\mathrm{srt}},\theta_{0}\right)=\beta_{0}+\beta_{1}x_{\mathrm{srt}}+\sum_{k=1}^{K}\delta_{k}{\left|x_{\mathrm{srt}}-\eta_{k}\right|}^{3},$$

where $\theta_{0}={\left(\beta_{0},\beta_{1},\delta_{1},\ldots ,\delta_{K}\right)}^{T}$, $\beta_{0}$ is the intercept, $\beta_{1}$ is the regression coefficient for time, $\delta =\left(\delta_{1},\ldots ,\delta_{K}\right)$ are random coefficients, $\eta_{k}$ are knots (i.e., the points where piecewise spline curves meet), and K is the total number of knots. Here, knot $\eta_{k}$ is the sample quantile of the $x_{\mathrm{srt}}$’s corresponding to the probability $k/\left(K+1\right)$ (Crainiceanu et al., 2005).

In the Scott Reef, the degree of disturbance exposure does not appear to be consistent across sites (see Figure S2 which shows the coral cover over time by site, and highlights the sites which appear to be most affected by the bleaching event in 1998). Hence, the inclusion of site specific/time‐varying covariates $z_{t}$ into the model (see below) should capture at least some of such variation. In addition, there could be variation between sites/reefs that cannot be explained by the available covariate information. In such cases, random effects can be included into the model as follows to capture such variation, where we note that sites are nested within reefs:

(2) $$\log\left(\frac{\pi_{\mathrm{srt}}}{1‐\pi_{\mathrm{srt}}}\right)=\gamma_{\mathrm{sr}}+\beta_{t}z_{t}+\beta_{d}d_{r}+f\left(x_{\mathrm{srt}},\theta_{0}\right),$$

where $\gamma_{\mathrm{sr}}$ represents the random effects that are assumed to follow $p\left(\gamma_{\mathrm{sr}}|\lambda_{r},\log\;\sigma_{s}\right)$ where $\lambda_{r}∼p\left(\lambda |\log\;\sigma_{r}\right)$, where p represents some distribution, and, $\log\;\sigma_{s}$ and $\log\;\sigma_{r}$ are the logarithm of the standard deviations of the site and reef random effects, respectively. Wood (2003) describes the extension of such a model to accommodate other potential covariates, and we follow this approach to incorporate reef level time‐varying covariates such as bleaching exposure and cyclone hours. In Equation (2), $z_{t}$ represents time‐varying covariates and $\beta_{t}$ is the corresponding vector of regression coefficients. Additionally, we incorporated three dummy variables to account for cyclone, severe cyclone, and bleaching exposures at different reef locations. The corresponding data matrix and the vector of regression coefficients are denoted as $d_{r}$ and $\beta_{d}$, respectively.

Coral cover is often impacted by disturbances such as cyclones and bleaching events, and some major events will result in sudden declines in coral cover trends (De'ath et al., 2012; Osborne et al., 2011). We propose that estimating the impact of the major bleaching event that occurred in 1998 can be achieved using an interrupted time series (ITS) regression approach (Bernal et al., 2017; Linden, 2015). The motivation for this is that, in general, an ITS approach can account for sudden changes in the trend due to some intervention introduced or disturbance that has occurred (McDowall et al., 2019). When applying ITS, the type of impact due to the disturbance should be hypothesized. This may include a gradual change in slope or in both the intercept and slope within the model for the mean response (Bernal et al., 2017). In addition, some disturbances may cause an immediate change in the trend while others may have a lag period before any effect is observed. The reader is referred to Bernal et al. (2017) for more details about modeling different types of sudden changes in time series data.

Based on hard coral cover trends over time (Figure S1), we hypothesized that the 1998 mass bleaching event resulted in changes to both the intercept and slope when modeling hard coral cover trajectories. Furthermore, it was proposed that the impact existed for years as mortality does not happen completely during or a few months after bleaching (Baird & Marshall, 2002; Gilmour & Smith, 2013). The model defined previously using Equation (2) can now be extended to accommodate such an impact as follows:

(3) $$\log\left(\frac{\pi_{\mathrm{srt}}}{1‐\pi_{\mathrm{srt}}}\right)=\gamma_{\mathrm{sr}}+\beta_{t}z_{t}+\beta_{d}d_{r}+f\left(x_{\mathrm{srt}},\theta_{0}\right)+\beta_{l}\mathrm{BLE}98_{\mathrm{srt}}+\beta_{s}\text{Time}98_{\mathrm{srt}},$$

where $\mathrm{BLE}98_{\mathrm{srt}}$ represents the bleaching impact, that is, $\mathrm{BLE}98_{\mathrm{srt}}=0$ before the bleaching event happened, and otherwise, it is equal to 1, and $\beta_{l}$ is the level change due to the bleaching impact. Here, $\text{Time}98_{\mathrm{srt}}$ represents the time before and after the bleaching event, that is, $\text{Time}98_{\mathrm{srt}}=0$ before the bleaching event occurred, and after that, time increases with survey time, and $\beta_{s}$ represents the corresponding slope change.

In the model, cyclone hours data were count values that varied over a large range; thus, the square‐root transformation (Weber, 1990) was applied before including this covariate into the model. This transformation was also applied to ensure a linear relationship was appropriate between cyclone hours and $\log\;\left(\pi_{\mathrm{srt}}/\left(1-\pi_{\mathrm{srt}}\right)\right)$ (O'Hara & Kotze, 2010). Previous studies have considered centering covariates to avoid numerical issues when fitting a given model, and we follow this approach for the time‐varying covariates (Selig et al., 2012; Vercelloni et al., 2014). Furthermore, we calculated $|x_{\mathrm{srt}}-\eta_{k}|$ by considering centered survey time (Crainiceanu et al., 2005).

Within a Bayesian framework, we are interested in estimating the joint posterior distribution $p\left(\theta ,\xi |y_{h},Z_{h},V_{h},d_{h}\right)$ of model parameters and random effects, where 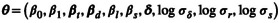 denotes all parameters in the model (Equation (3)), ξ is a matrix representation for the nested random effects, $d_{h}$ denotes previous surveys at Scott Reef, $V_{h}$ represents data matrices related to $|x_{\mathrm{srt}}-\eta_{k}|$ and the ITS component (i.e., $\mathrm{BLE}98_{\mathrm{srt}}$ and $\text{Time}98_{\mathrm{srt}}$), $y_{h}$ denotes the previously collected hard coral cover data (i.e., $y_{\mathrm{srt}},s=1,2,3;r=1,\ldots ,7;t=1994.83,\ldots ,2016.08$), and $Z_{h}$ are the previously collected time‐varying covariates (for times 1994.83 to 2016.08) where we have shifted notation such that all historical data will now be indexed by h. This will be convenient when considering future monitoring scenarios later in the paper. To estimate the posterior distribution (see Appendix S3 for more details), Markov Chain Monte Carlo (MCMC) methods can be used. For this purpose, WinBUGS was implemented (Lunn et al., 2000).

To find the most appropriate model to describe the historical data at Scott Reef, we considered the ℳ–closed perspective of Bernardo and Smith (2009). Accordingly, the most appropriate model for the data is assumed to be contained within a finite set of L candidate models indexed by $m\in \left\{1,2,\ldots ,L\right\}$. We defined the class of models by considering the following components: the nested random effects for sites within reef locations (NRE); the smooth component (SC); and all available covariates (ALL COV), that is, Time, Bleaching, Cyclone hours, Interrupted 98 (i.e., $\mathrm{BLE}98_{\mathrm{srt}}$ and $\text{Time}98_{\mathrm{srt}}$), location‐specific covariates impacts, that is, Cyclone Loc2 (i.e., Cyclone Loc and Severe cyclone Loc) and Bleaching Loc, and the interaction between Bleaching and Cyclone. The most appropriate model within this class was then determined via the deviance information criterion (DIC) with a preference for the model with the smallest of these values (Spiegelhalter et al., 2014). Prior information was specified to be vague on likely range of values of each parameter (Table S3). In addition, to appropriately capture the nonlinear features of the data, a specific number of knots needs to be determined. For this, we followed the approach of Ruppert (2002) where the number of knots was increased until there was little to no improvement in model fit. This resulted in the use of three knots.

#### 5.1.2 Form prior for design

As the above model will be fitted within a Bayesian inference framework, a posterior distribution of the parameters will be obtained. Such a posterior distribution quantifies the uncertainty about the model parameters given the historical data, and it is this distribution that is used to form prior information for design. That is, this posterior distribution becomes the prior distribution for design such that any additional data that are collected in future monitoring will update this prior information which will presumably reduce uncertainty about the parameters. It is this reduction in uncertainty (or relative gain in information) that is used to guide future sampling. In particular, this can be used to evaluate a design with respect to addressing a given monitoring objective, and this is described in the next section.

### 5.2 Assessing designs

This section explains how to evaluate designs in terms of achieving a certain monitoring objective (Figure 2, middle) based on prior information that has been obtained from historical data. A general approach is adopted through a utility function which is constructed to encapsulate the monitoring objective. A design is then selected, so that this objective is expected to be optimized.

#### 5.2.1 Propose design

Define a design as $d={\left(d_{1},d_{2},\ldots ,d_{n_{s}}\right)}^{t}$, where $n_{s}$ is the number of sites appearing in a proposed sampling design out of all sites (i.e., 7 reef locations × 3 sites = 21 sites). The usefulness of such a design d can be quantified via what is called a utility function which evaluates how much information will be provided from data y to address a specific monitoring objective. As it is unknown what data will be observed, the expectation of the utility function is taken with respect to this and other unknowns as follows:

(4) $$E\left[u\left(d,z,y|y_{h},Z_{h},V_{h},d_{h}\right)\right]=\int_{ϒ}u\left(d,z,y|y_{h},Z_{h},V_{h},d_{h}\right)p\left(y|z,d,y_{h},Z_{h},V_{h},d_{h}\right)dy,$$

where z represents specific values of the time‐varying covariates which define particular future monitoring scenarios. Further details about these scenarios will be provided later in this section.

The choice of a utility function depends on the monitoring objective. Here, our goal is to determine the relative importance of survey locations for providing information about coral health based on a statistical model, so we consider gathering as much information as possible about the parameters in this model as the monitoring objective. Accordingly, a variety of parameter estimation utility functions could be considered including the Kullback–Leibler divergence (KLD) and a Bayesian version of the D‐optimality criterion. We chose KLD (Kullback & Leibler, 1951) as we are broadly interested in the precise estimation of all parameters with respect to the prior information. The KLD utility function can be expressed as follows (Friel & Pettitt, 2008):
(5)

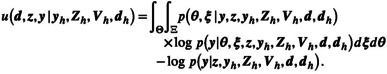

Evaluating this utility measures how much the posterior distribution diverges from the prior. In terms of designs, a larger deviation for a given design d indicates more has been learned from data collected. Thus, we seek a design d that maximizes the expectation given in Equation (4) where the utility is defined in Equation (5).

#### 5.2.2 Approximate the expected utility

To find an optimal design, we need to evaluate the expected utility. However, typically, this expression does not have a closed‐form solution. Thus, a numerical approximation is required. For this, one can use Monte Carlo integration (Ryan, 2003) which can be defined as follows:

(6) $$\begin{array}{l} E\left[u\left(d,z,y|y_{h},Z_{h},V_{h},d_{h}\right)\right]\approx \frac{1}{J}\sum_{j=1}^{J}u\left(d,z,y^{\left(j\right)}|y_{h},Z_{h},V_{h},d_{h}\right), \end{array}$$

where *J* (≥100) is the controlling parameter for Monte Carlo integration. This approach to approximate the expected utility is outlined in Algorithm 1, which begins by initializing some parameters (line 1). To approximate the expected utility for a given design d, many datasets need to be simulated based on the given design (line 2). For this purpose, we simulate parameter and random effect values from the prior distribution (line 3). To simulate hard coral cover for the next survey time point t (line 4), we propose a Taylor series expansion around the mean at the last survey point, that is, 2016.08 (Table S7). In such a case, a bivariate Taylor series expansion needs to be applied as the regression model includes two time variables following the incorporation of the interrupted regression component. For the given model, the Taylor series approximation can be described as follows: $f\left(x_{\mathrm{srt}},\text{Time}98_{\mathrm{srt}}\right)\approx f\left(a,b\right)+f_{x_{\mathrm{srt}}}\left(a,b\right)\left(x_{\mathrm{srt}}-a\right)+f_{\text{Time}98_{\mathrm{srt}}}\left(a,b\right)\left(\text{Time}98_{\mathrm{srt}}-b\right)$, where $f\left(x_{\mathrm{srt}},\text{Time}98_{\mathrm{srt}}\right)$ is the value of the function at the next survey point (i.e., t = 2016.33) and $\left(a,\;b\right)$ are values which with the Taylor series is centered (see Appendix S7 for extrapolation results). The posterior distribution needs to be approximated for each simulated dataset (line 5). Given this needs to be performed a large number of times, this is a computationally demanding step in the algorithm. To address this, a Laplace approximation (Overstall et al., 2018) was adopted. Given this approximation to the posterior distribution, the KLD utility can be evaluated (see Appendix S9 for more details) (lines 6–7). Finally, an average of KLD utility values is used to approximate the expected utility (line 9).

ALGORITHM 1 **Approach to approximate the expected utility**

**Table:** 

1. Initialise $d,y_{h},Z_{h},V_{h},d_{h},z,t,J$
2. For j=1 to J do
3. Simulate $\theta^{\left(j\right)},\xi^{\left(j\right)}∼p\left(\theta ,\xi |y_{h},Z_{h},V_{h},d_{h}\right)$
4. Simulate $y^{\left(j\right)}∼p\left(y|\theta^{\left(j\right)},\xi^{\left(j\right)},z,y_{h},Z_{h},V_{h},d,d_{h}\right)$ at the next survey time t based on d via a Taylor series approximation to the mean response
5. Estimate $p\left(\theta ,\xi |y,z,y_{h},Z_{h},V_{h},d,d_{h}\right)$ via Laplace approximation
6. Evaluate KLD utility $u\left(d,z,y^{\left(j\right)}|y_{h},Z_{h},V_{h},d_{h}\right)$
7. Store $u\left(d,z,y^{\left(j\right)}|y_{h},Z_{h},V_{h},d_{h}\right)$
8. End for
9. Output $E\left[u\left(d,z,y|y_{h},Z_{h},V_{h},d_{h}\right)\right]\approx \frac{1}{J}\sum_{j=1}^{J}u\left(d,z,y^{\left(j\right)}|y_{h},Z_{h},V_{h},d_{h}\right)$

### 5.3 Optimization and evaluation of the design

This section describes the third stage of our Bayesian adaptive design framework: optimization and evaluation of the design (Figure 2, right). The procedure used for this optimization is described next, along with the design formulation, the selection of future disturbance scenarios and a so‐called design efficiency which is used to evaluate designs.

#### 5.3.1 Optimize design

Using Algorithm 1, we are now able to approximate the expected utility of a given design d. The next step is to find the optimal design $d^{*}$ out of the set of candidate designs which maximizes the expected utility, that is, $d^{*}=\underset{d}{\arg\;\max}\;E\left[u\left(d,z,y|y_{h},Z_{h},V_{h},d_{h}\right)\right]$. Here, candidate designs d need to be formulated in accordance with monitoring scenarios, and these were constructed on the basis of likely sampling problems that could be encountered on Scott Reef into the future. For Objective (i), we will investigate whether some reef locations within Scott Reef have greater utility than others. Accordingly, seven candidate designs were formulated considering all possible combinations where six of seven reef locations will be sampled. The corresponding designs were labeled as SL1, …, SS3 where, for example, design SL1 denotes that no data will be collected from the reef location SL1 for the next survey time. As there will be a relatively small number of potential candidate designs (i.e., seven) under this objective, we will enumerate all seven designs to determine the optimal. Next, under Objective (ii), we determine the optimal design consisting of the most informative site (out of the three) at each reef location. To locate these optimal designs, the coordinate‐exchange algorithm was used based on five randomly selected initial designs (Meyer & Nachtsheim, 1995). Here, the coordinate‐exchange algorithm was implemented as there are actually many potentially optimal designs to assess, so applying an exhaustive search would not be computationally efficient.

#### 5.3.2 Evaluate design within monitoring scenario

To evaluate our adaptive designs, two disturbance scenarios (i.e., two different values for z) were considered. These were: (a) actual covariate data collected at the next survey time (i.e., 2016.33) and (b) bleaching and cyclone impacts including an interaction between them at each reef location (see Appendix S8 for more details). This means that Scenario (a) withdraws the prevailing cyclone exposure while Scenario (b) includes cyclone location disturbances and cyclone‐bleaching interactions for each reef location. Each objective defined previously will be assessed under these two disturbance scenarios.

To evaluate the optimal designs that will be found, we will evaluate the amount of information that is expected to be obtained, and compare this to the amount of information that would be obtained if data were collected at all sites/reefs. For this comparison, a design efficiency can be evaluated as follows:

(7) $$\begin{array}{l} \mathrm{Eff}=\frac{E\left[u\left(d,z,y|y_{h},Z_{h},V_{h},d_{h}\right)\right]}{E\left[u\left(d_{L},z,y|y_{h},Z_{h},V_{h},d_{h}\right)\right]}, \end{array}$$

where $E\left[u\left(d,z,y|y_{h},Z_{h},V_{h},d_{h}\right)\right]$ and $E\left[u\left(d_{L},z,y|y_{h},Z_{h},V_{h},d_{h}\right)\right]$ are evaluations of the approximate expected utility (Equation (6)) for a design d and the design $d_{L}$ (which denotes that all sites/reefs are included in the next survey time), respectively. When evaluating this efficiency, to reduce the impact of the stochastic approximation to the expected utility, the evaluation will be repeated 20 times (independently) for d and $d_{L}$, and the efficiency will be evaluated each time. The mean of these 20 efficiencies will then be taken as the design efficiency.

## 6 RESULTS

### 6.1 Quantifying prior information

To select the most appropriate model for the Scott Reef hard coral cover data, we defined a class of models by considering all components described in Section 5.1. The corresponding model comparison results based on DIC are provided in Table S4. As the model with a smaller value of DIC is preferred, the most appropriate model found for hard coral cover can be described as follows:
(8)

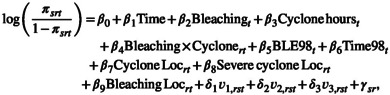

where $\beta_{i},i=1,\ldots ,9$ are the regression coefficients and $\delta_{1}$, $\delta_{2}$, and $\delta_{3}$ are random coefficients. The goodness‐of‐fit of this model was assessed and found to be appropriate, see Appendix S6 for further details. Then, the posterior distribution of this model was used as the prior distribution for all subsequent design evaluations.

### 6.2 Optimization and evaluation of the design

#### 6.2.1 Importance of reef locations

**FIGURE 3:**
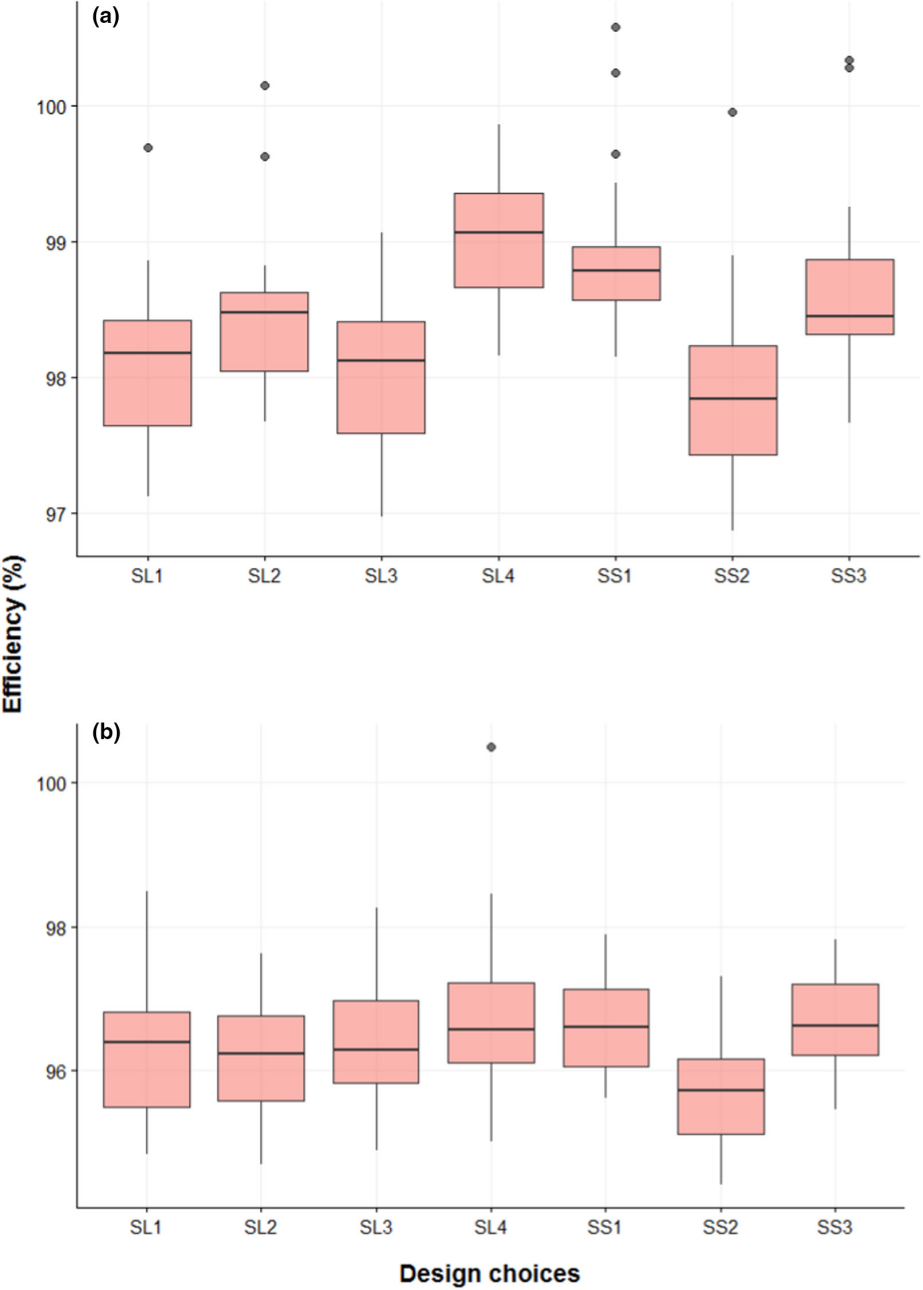
Utility evaluation results under disturbance Scenario (a) and (b) for seven designs (*x*‐axis). *y*‐axis represents design efficiency of each design when compared to sampling all seven reef locations.

Under Objective (i), we aim to determine the relative importance of seven reef locations at Scott Reef. First, disturbance Scenario (a) was considered. For this evaluation, we formulated seven designs (i.e., SL1, …, SS3), as described in Section 5.3.1. To evaluate these designs, the KLD expected utility was evaluated. These results (as design efficiencies) are shown in Figure 3a, where the efficiency is with respect to the design where all reef locations are sampled. A summary of the utility evaluations is given in Table 1 to aid in interpretation.

**TABLE 1:** Summary of utility evaluations for seven designs under Scenario (a) in Objective (i).

Design	Mean efficiency (%)	Standard deviation
SL1	98.14	0.59
SL2	98.46	0.60
SL3	98.01	0.52
SL4	99.03	0.46
SS1	98.92	0.62
SS2	97.93	0.71
SS3	98.68	0.68

According to Table 1, SL4 is the optimal design as it has the highest mean efficiency. As the missing reef location within this design is SL4, this reef location can be considered as the least informative reef location under Scenario (a). This suggests that less information is expected to be lost by omitting reef location SL4 compared with omitting any other reef location. Similarly, the reef location SS2 can be reported as the most informative reef location as omitting this reef location resulted in the largest reduction in efficiency (Table 1). However, it should be noted that there is very little difference in the efficiency values between these designs (Figure 3a). Indeed, some of the differences observed could potentially be due to Monte Carlo error.

Second, we evaluated Objective (i) under the disturbance Scenario (b). The corresponding design efficiencies and expected utility values are provided in Figure 3b and Table 2, respectively. It is evident from Figure 3b that all the reef locations have similar median efficiency values under Scenario (b) except for SS2, which aligns with the results under Scenario (a). The design SL4 has the highest efficiency; thus, the reef location SL4 can be reported as the least informative reef location under Scenario (b). Furthermore, comparing Figure 3a,b shows that overall there are similar efficiencies for designs under the two disturbance scenarios considered. This suggests that the optimal design is robust to the two scenarios considered under Objective (i).

**TABLE 2:** Summary of utility evaluations for seven designs under Scenario (b) in Objective (i).

Design	Mean efficiency (%)	Standard deviation
SL1	96.29	1.05
SL2	96.16	0.85
SL3	96.36	0.88
SL4	96.78	1.19
SS1	96.60	0.68
SS2	95.73	0.80
SS3	96.64	0.68

To explore these design selections, consider that the posterior means will be similar to the prior means, so this should not particularly contribute to the optimal design selection under KLD utility function, see Equation S1. Accordingly, these design selections could be driven by the posterior variance–covariance of the parameters. Upon investigating this, the larger utility values appeared to be related to the estimation of the reef random effect standard deviation parameter (i.e., log $\sigma_{r}$). This is typically where the largest change from the prior was observed, and thus could potentially be driving the design selection, that is, designs that provide more information on the variability of coral cover between reef locations are being preferred.

#### 6.2.2 Informative sites at each reef location

Under Objective (ii), we determined the optimal design that consists of the most informative site at each reef location subject to two disturbance scenarios. The selected sites from each reef location into the optimal designs under the two scenarios are reported in Table S8. It can be seen from the table that the optimal designs contain different site combinations under the two scenarios. It was found through investigating these optimal designs that the estimation of log $\sigma_{r}$ again appeared to be a main contributor to these optimal design selections. This indicates that the optimal site combination under a given disturbance scenario provides more information about the between reef variability.

The optimal designs under the two scenarios have mean efficiencies of 86.28 (5.49)% and 71.18 (2.62)%, respectively. These reductions in mean efficiency indicate that if disturbances are similar to previous years, then minimal sampling (i.e., one site per reef location) will capture a substantial proportion of information. However, when a variety of disturbance combinations are observed, then there appears to be more information lost, so there might be value to undertaking additional sampling in such cases.

## 7 DISCUSSION

This study developed adaptive design methods using semiparametric and ITS models through utilizing information captured through such modeling to guide future surveys at Scott Reef. We demonstrated the use of such a modeling approach in finding adaptive design when data showed nonlinear trends with some sudden shifts over time. For this purpose, it was shown that changes around major environmental disturbances in ecological monitoring could be accounted for using an ITS regression modeling approach. This enabled prior information from historical data to be appropriately formed when such data potentially exhibit complex ecological relationships.

We assessed the importance of reef locations under Objective (i) subject to two disturbance scenarios. The results showed that there was little difference between the selection of which reef location to omit under either scenario. This indicates that the design choice is relatively inconsequential. Also, dropping one reef location resulted in little information loss, allowing the survey effort to be reduced without losing a substantial amount of information about the parameters in the developed model.

Under Objective (ii), we found optimal designs consisting of one site at a given reef location based on two disturbance scenarios. This provided insight into the most appropriate site to sample from a given reef location depending on prevailing disturbance conditions. The differences between the optimal designs between these two scenarios suggest that site selection depends on the disturbances that have been observed, and our methods provide a framework with which to make this assessment. Additionally, it appears that the design choices made under Objectives (i) and (ii) are associated with the estimation of the reef random effect standard deviation parameter (i.e., log $\sigma_{r}$) meaning that we are learning about how coral cover varies between reefs.

In terms of modeling monitoring data, the Gompertz model has been considered recently (MacNeil et al., 2019) for capturing nonlinear relationships in population growth. However, such a model proved to not be flexible enough to capture nonlinear trends where observations have been collected with unequal time gaps. In such circumstances, semiparametric modeling approaches can be utilized to capture nonlinear trends, as demonstrated in this study. The consideration of such a modeling approach meant that new design methods needed to be proposed such that adaptive designs could be found in this context. This was demonstrated by considering two future disturbance scenarios, and assessing the performance of these designs against more resource intensive sampling.

It is important to note that the adaptive designs found in this work could potentially change depending on the prior information that was obtained from the historical data. This is why significant effort was invested in assessing the goodness‐of‐fit of the model through cross‐validation in addition to the standard approach of assessing the posterior predictive distribution. Given this, we suggest it is crucial that the exact analysis plan for evaluating the monitoring objective be encoded into the utility function. That is, in this work, the health of Scott Reef will be assessed through hard coral cover as estimated from the developed hierarchical model. In such a case, the design found is optimal. In contrast, if some other approach will be used to assess the health of Scott Reef, then there is no guarantee that the proposed designs will be efficient or expected to provide maximum information.

In terms of extending our model, a number of options could be considered including incorporating the effects of temperature on coral health. For this, we would like to note that bleaching is a better direct measure of the impact of temperature on coral, so additionally having temperature would most likely be redundant. Similarly, one could consider incorporating the effects of increasing acidification of the ocean. However, such impact would likely be minor and therefore will most likely be swamped by the impact of cyclone and bleaching events. Furthermore, such effects would not be expected to change rapidly enough in space and/or time to be relevant to design considerations. Other extensions that could be considered include further capturing delayed effects of coral bleaching (see Graham et al., 2007) which may improve the model fit in, for example, the final sampling year where some discrepancy is observed at the initial stages of a bleaching event, see Figure S3. Depending on how this is captured, sampling designs could potentially be developed to estimate such delays.

The use of flexible semiparametric modeling approaches as adopted in this paper can lead to a statistical model that is overfitted. In our case, this flexibility (and therefore potential for overfitting) is largely controlled by the number of knots in the model. Ideally, there would be enough knots to effectively capture trends in the data but not so many that random variations are being explained. Automatic procedures are available which provide a choice for the number of knots (e.g. Ruppert, 2002) where one should effectively penalize overly complicated models. In our work, we followed the approach of Ruppert (2002) which led to a choice of three knots. Such a low number mitigates overfitting but still provides flexibility in describing trends in data. In addition, cross‐validation approaches were adopted to assess model fit which should provide further mitigation against overfitting.

In terms of future research directions, there is interest in the transferability of our designs and our adaptive design approach to other environmental monitoring programs. Broadly, we suggest that, in principle, the approach considered in this work could be applied within a variety of monitoring programs where data collection is expensive, and therefore, the use of resources to collect data needs careful consideration and planning. In order for this approach to be applied in such settings, one needs a quantifiable monitoring objective defined based on a planned analysis. This is critical as the analysis defines the uncertainty in the monitoring objective, and the reduction in this uncertainty is used to compare and optimize sampling effort. In terms of the transferability of the actual designs, we note that the designs are model‐based so some insight into transferability may be provided from a modeling prospective (e.g., Yates et al., 2018), particularly general learnings about what sampling is informative to achieve a given objective. However, further exploration of this area is needed before anything concrete can be concluded.

It is expected that disturbances will increase in the future; both in terms of frequency and in terms of severity. We note that Scott Reef has experienced multiple major and moderate bleaching and cyclone disturbance events within the time period considered in this paper, so significant disturbance events are not completely out of consideration in this work. Accordingly, we suggest that the recommendations we have made for future monitoring are still relevant. In particular, within the data we have analyzed, we have seen that increased severity of disturbance reduces variability among sites suggesting that the impact is more consistent across an entire reef location. Given this, the finding that no particular site needs to be preferred in future sampling would seem reasonable, and this is what was found when we evaluated our adaptive designs.

## 8 RECOMMENDATIONS FOR FUTURE REEF MONITORING BASED ON FINDINGS FROM SCOTT REEF

- Pre‐assessment of the expected information gain by location (e.g., site or reef location) can be used to determine whether any locations can be prioritized for data collection. Here, it was found that information gain from sites was similar, so no particular location needed to be prioritized over another.
- After an extensive monitoring period, explore reduced sampling practices as there is potential to reduce sampling effort (e.g., drop site or reef location) without experiencing significant information loss about coral health.
- Evaluate disturbance patterns at monitoring locations as these can influence information gain, for example, here, it was shown that more information about coral health was obtained when new disturbance patterns were experienced when compared to historical disturbance patterns.
- On‐going review of monitoring practices is recommended to assess effectiveness of adaptive designs.

## AUTHOR CONTRIBUTIONS

**Pubudu Thilan Abeysiri Wickrama Liyanaarachchige:** Conceptualization (lead); formal analysis (lead); investigation (lead); methodology (lead); project administration (lead); software (lead); validation (lead); visualization (lead); writing – original draft (lead). **Rebecca Fisher:** Conceptualization (lead); validation (equal); writing – review and editing (equal). **Helen Thompson:** Conceptualization (equal); supervision (equal); writing – review and editing (equal). **Patricia Menendez:** Conceptualization (equal); supervision (equal); writing – review and editing (equal). **James Gilmour:** Conceptualization (equal); data curation (equal); validation (equal). **James M. McGree:** Conceptualization (lead); funding acquisition (lead); methodology (lead); project administration (lead); supervision (lead); validation (equal); writing – review and editing (lead).

## CONFLICT OF INTEREST

The authors declare no conflicts of interest.

## Supporting information


Appendix S1–S10

## Data Availability

Access to the data for this study to reproduce the results is available through the following link: https://doi.org/10.25845/asfw‐dz73.
