# Supplementary material for: Adaptive monitoring of coral health at Scott Reef where data exhibit nonlinear and disturbed trends over time
Source: Ecol Evol. 2022 Sep 11;12(9):e9233. doi: 10.1002/ece3.9233 (PMC9465202; doi:10.1002/ece3.9233)
Supplement: Supplementary file 1 — Appendix S1–S10 [file ECE3-12-e9233-s001.pdf]

Appendix A

A.1. Scott Reef surveys

In Scott Reef, there are three nested sites at each reef location and one observation has been collected from each site for a given survey time. The number of samples collected for each survey time at Scott Reef is summarised in Table A.1. For example, 21 observations have been collected for survey time 1994.83 using three sites at each reef location. It can be seen from the table that there are some variations in survey practices over time. In particular, site replication has been abandoned after the bleaching event in 2016.

A.2. Distribution of hard coral cover

We visualise the distribution of coral cover at Scott Reef to see how it varies over time (Figure A.1). Accordingly, it exhibits a nonlinear trend with a sudden shift around the 1998 bleaching event. In addition, the distribution of hard coral cover by reef locations is shown in Figure A.2. The years of cyclone events be provided Table A.2 . It can be seen from the individual figures that different reef locations have been impacted differently due to bleaching and cyclone disturbances.

A.3. Posterior estimation

The joint posterior distribution of model parameters and random effects can be expressed in a Bayesian framework as follows: $p(\boldsymbol{\theta}, \boldsymbol{\xi} \mid \boldsymbol{y}_h, \boldsymbol{Z}_h, \boldsymbol{V}_h, \boldsymbol{d}_h) \propto$

Table A.1: Survey variations over time at Scott Reef. The first column represents survey times used at Scott Reef where the decimal places indicate the survey times within the given year. The next three columns represent three sites at each reef location. The value seven for a particular site index means that the corresponding site has been surveyed at all the seven reef locations. The last column represents the total number of observations collected over the seven reef locations using three sites which are located at each reef location. Starting from 2016.08, only the first site has been surveyed at each reef location which results in the total number of seven observations for the corresponding survey times.

	Site index			
Survey times	1	2	3	Total number of observations
1994.83	7	7	7	21
1995.83	7	7	7	21
1996.83	7	7	7	21
1997.92	7	7	7	21
1998.58	3	0	1	4
1998.90	7	7	7	21
1999.92	6	5	5	16
2001.90	6	6	6	18
2004.00	6	6	6	18
2004.33	3	0	1	4
2004.83	7	7	0	14
2008.08	7	7	7	21
2010.83	7	7	7	21
2012.92	7	7	7	21
2014.83	7	6	7	20
2016.08	7	0	0	7
2016.33	7	0	0	7
2016.83	7	0	0	7
2017.92	7	0	0	7

$p(\boldsymbol{y}_h \mid \beta_0, \beta_1, \boldsymbol{\beta}_t, \boldsymbol{\beta}_d, \beta_l, \beta_s, \gamma_{sr}, \boldsymbol{\delta}, \boldsymbol{Z}_h, \boldsymbol{V}_h, \boldsymbol{d}_h) p(\gamma_{sr}|\lambda_r, \log\sigma_s) p(\lambda_r|\log\sigma_r) p(\boldsymbol{\delta}| \log\sigma_{\boldsymbol{\delta}}) p(\boldsymbol{\theta})$ where the first term on the right hand side is the conditional likelihood (i.e. conditional on the random effects), and then, $p(\boldsymbol{\delta}|\log\sigma_{\boldsymbol{\delta}})$ is the distribution of random coefficients, and $p(\boldsymbol{\theta})$ is the prior distribution (Table A.3). In this study, all historical data at Scott Reef were analysed within a

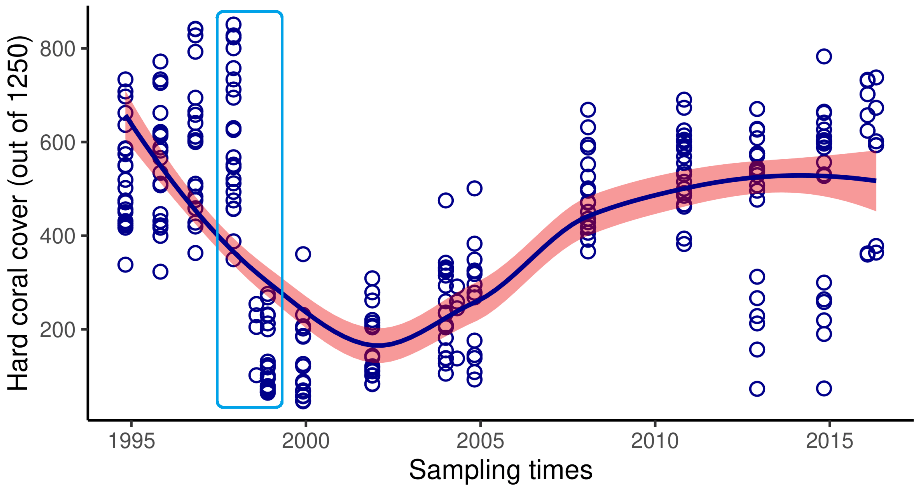

Figure A.1: Distribution of hard coral cover at Scott Reef over time. The fitted blue line is the mean curve produced using *geom_smooth* function in ggplot2 package in R using the default smooth method (i.e. "loess"). The shaded red area represents the corresponding 95% confidence interval for the mean. The sudden shift in hard coral cover due to 1998 bleaching event is marked using a light blue colour box.

Table A.2: The years of reef location specific cyclone events. Here, C represents Cyclone or multiple cyclones and SC represents Severe Cyclone.

From	To	SL1	SL2	SL3	SL4	SS1	SS2	SS3
1994.83	1995.83							
1995.83	1996.83			C				
1996.83	1997.92			C	C			
1997.92	1998.90							
1998.90	1999.92							
1999.92	2001.90			C				
2001.90	2004.00							
2004.00	2004.83		C			SC	C	
2004.83	2008.08							
2008.08	2010.83							
2010.83	2012.92	C		SC	SC			
2012.92	2014.83							
2014.83	2016.08							
2016.08	2016.83							

Bayesian framework using WinBUGS to sample from the posterior distribution for the purpose of model selection.

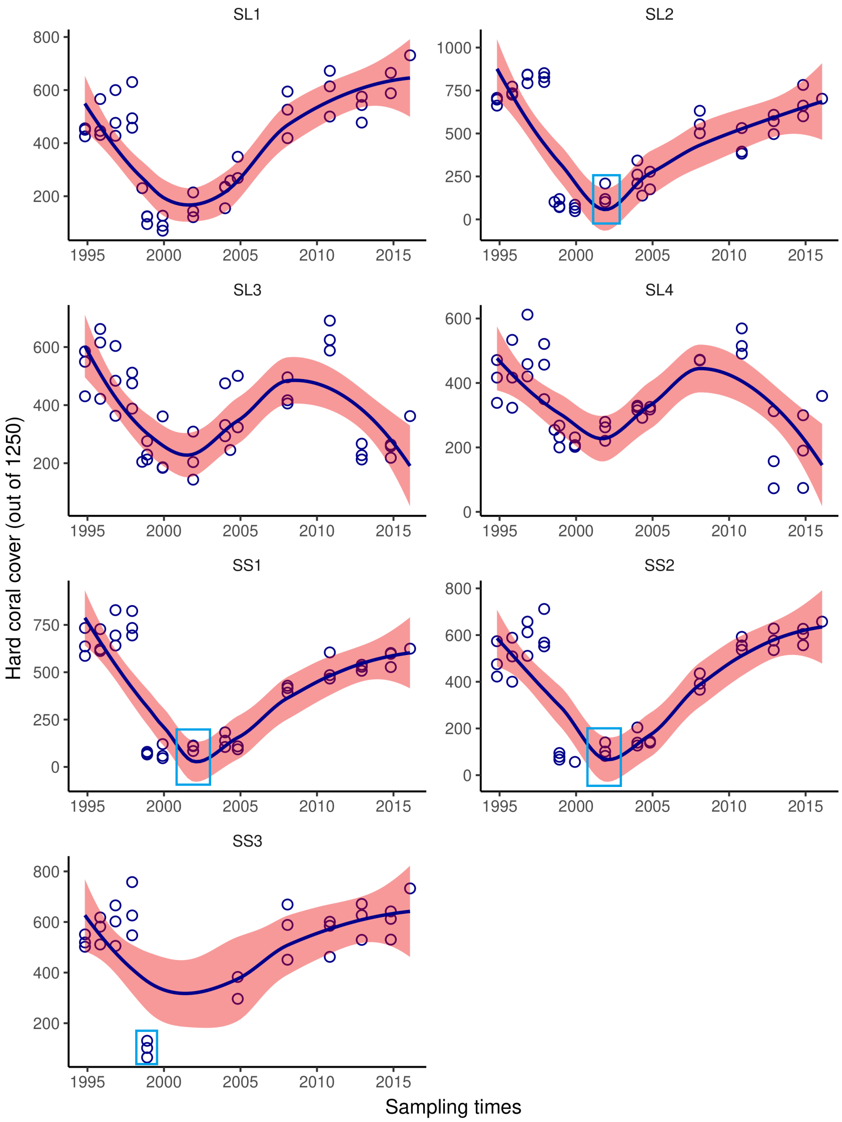

Figure A.2: Distribution of hard coral cover by reef locations at Scott Reef. The fitted blue lines are the mean curves produced using *geom_smooth* function in ggplot2 package in R using the default smooth method (i.e. "loess"). The shaded red areas represent the corresponding 95% confidence intervals for the means. Some badly affected reef locations due to 1998 bleaching event are marked using light blue colour boxes. Here, the impact of a bleaching event can last for years after the event so some locations have been highlighted after the year 1998.

Table A.3: The individual components of the adopted prior distribution $p(\boldsymbol{\theta})$.

**Parameters**	**Prior distributions**
Each component of $\boldsymbol{\beta}_t$ and $\boldsymbol{\beta}_d$	$\mathrm{N}(0, 100^2)$
Regression coefficients β_0, β_1, β_l, and β_s	$\mathrm{N}(0, 100^2)$
Random coefficients δ_k	$\mathrm{N}(0, \sigma_\delta^2)$
Random effects for reef locations λ_r	$\mathrm{N}(0, \sigma_r^2), r = 1, \ldots, 7$
Random effects for sites γ_{sr}	$\mathrm{N}(\lambda_r, \sigma_s^2), r = 1, \ldots, 7; s = 1, 2, 3$
linear log σ_δ	$\mathrm{N}(5, 1)$
Reef random effects log σ_r	$\mathrm{N}(-1, 1)$
Site random effects log σ_s	$\mathrm{N}(-1, 1)$

A.4. Model selection

We used the $\mathcal{M}$-closed perspective to determine the most appropriate model to describe the historical data at Scott Reef. The model selection results using DIC criterion for the formulated models is provided in Table A.4.

Table A.4: Model selection results using DIC criterion. The "+" and "-" imply whether respective component/covariate is included or excluded from the model. The components and covariates dropped at each stage are shown using bold text.

**Components in the model**	**DIC**
ALL COV+NRE+SC	8897.710
ALL COV+NRE-**SC**	9343.130
ALL COV-**NRE**+SC	11525.400
ALL COV-**NRE-SC**	11998.300
ALL COV+NRE+SC **-interaction-Bleaching Loc-Cyclone Loc2**	17367.400
ALL COV+NRE+SC **-interaction-Bleaching Loc-Cyclone Loc2-Intterruppted98**	27588.400
ALL COV +NRE+SC **-interaction-Bleaching Loc-Cyclone Loc2-Intterruppted98-Cyclone hours**	27686.800
ALL COV+NRE+SC **-interaction-Bleaching Loc-Cyclone Loc2-Intterruppted98-Cyclone hours-Bleaching**	28061.200
Null model	28742.200

A.5. Posterior summary

The posterior summary statistics including 95% credible intervals are reported in Table A.5, for the final model choice. Accordingly, all parameters are significant (i.e. credible intervals do not contain zero) except the coefficients for Bleaching. This Bleaching covariate represents bleaching occurrences except for the mass bleaching event in 1998 that was accounted for using ITS approach. Except for that mass bleaching event, Scott reef has undergone only a few bleaching events. As Bleaching is an indicator variable in the model, it may be that bleaching present does not contrast with the bleaching absent; thus, it could result in a non-significant bleaching effect. Furthermore, it should be noted that bleaching is involved in a significant interaction with cyclones in the model. Thus, this disturbance is having a significant effect depending upon the value of the cyclone variable. In terms of random effects, the posterior distribution summary is provided in Table A.6. Accordingly, all reef random effects are non-significant. This indicates that there is not a lot of variability in hard coral cover trajectories at the reef location level. However, there are some significant site random effects indicating that there are distinguishable hard coral cover trajectories at the site level.

A.6. Goodness-of-fit

To assess the goodness-of-fit of the model defined above, a posterior predictive check was used. This involved comparing the observed data with data

Table A.5: Summary of the posterior distribution of the model parameters.

Parameter	Mean	Standard deviation	Lower bound of 95% credible interval	Upper bound of 95% credible interval
Log σ_δ	1.150	0.490	0.360	2.218
Log σ_r	-1.992	0.476	-2.969	-1.122
Log σ_s	-1.704	0.192	-2.054	-1.304
Intercept	0.656	0.093	0.470	0.826
Time	-9.657	0.711	-11.000	-8.107
Bleaching	0.032	0.025	-0.017	0.080
Cyclone hours	-0.038	0.002	-0.042	-0.035
BLE98	-1.649	0.026	-1.700	-1.598
Time98	7.216	0.448	6.233	8.068
Cyclone loc	-0.418	0.014	-0.444	-0.391
Severe cyclone loc	-1.426	0.022	-1.469	-1.382
Bleaching loc	-1.010	0.019	-1.047	-0.973
Bleaching × Cyclone	-0.331	0.022	-0.376	-0.288
Random coefficient 1	-2.113	0.167	-2.433	-1.751
Random coefficient 2	1.092	0.059	0.976	1.207
Random coefficient 3	2.821	0.145	2.522	3.089

simulated from the posterior predictive distribution (Gelman and Hill, 2007). This comparison is shown in Figure A.3 where the derived model appears to describe the trends and variability in the data well. Similarly, we checked the posterior predictive distribution at each of the seven reef locations (Figure A.4). From the individual figures, it can be seen that the trends and variability of the data are well captured by the model at the reef location level.

A.7. Extrapolation results

To investigate the appropriateness of the proposed (Graham et al., 2007) Taylor series approximation to the mean response, this approximation was

Table A.6: Summary of the posterior distribution of the random effects. The first row represents random effects related to the seven reef locations. The second row represents random effects related to the 21 sites locations nested within reef locations.

Random effect	Mean	Standard deviation	Lower bound of 95% credible interval	Upper bound of 95% credible interval
SL1	-0.0150	0.1001	-0.2146	0.1917
SL2	0.1848	0.1251	-0.0254	0.4585
SL3	-0.0139	0.1005	-0.2133	0.1903
SL4	-0.1195	0.1090	-0.3546	0.0764
SS1	0.0445	0.1029	-0.1523	0.2631
SS2	-0.0824	0.1046	-0.3053	0.1133
SS3	0.0287	0.1019	-0.1609	0.2441
SL1-1	0.0533	0.0803	-0.0958	0.2235
SL1-2	-0.0639	0.0806	-0.2133	0.1074
SL1-3	-0.0724	0.0808	-0.2238	0.0979
SL2-1	0.1278	0.0805	-0.0209	0.2982
SL2-2	0.4847	0.0807	0.3345	0.6561
SL2-3	0.2874	0.0806	0.1377	0.4586
SL3-1	-0.0450	0.0805	-0.1959	0.1251
SL3-2	0.3075	0.0805	0.1579	0.4801
SL3-3	-0.3332	0.0807	-0.4847	-0.1618
SL4-1	-0.2825	0.0805	-0.4321	-0.1120
SL4-2	-0.0817	0.0805	-0.2325	0.0887
SL4-3	-0.2322	0.0807	-0.3836	-0.0618
SS1-1	-0.0068	0.0805	-0.1548	0.1646
SS1-2	0.1826	0.0804	0.0322	0.3527
SS1-3	0.0349	0.0807	-0.1163	0.2038
SS2-1	-0.1777	0.0805	-0.3264	-0.0064
SS2-2	-0.0882	0.0804	-0.2383	0.0817
SS2-3	-0.1483	0.0810	-0.2990	0.0222
SS3-1	0.0284	0.0808	-0.1212	0.1978
SS3-2	0.2382	0.0807	0.0892	0.4092
SS3-3	-0.1311	0.0814	-0.2816	0.0417

used to predict future but known hard coral cover in our data set (akin to a leave-one-out cross validation approach), we visualised the extended curves for each of the seven reef locations. For instance, Figure A.5 shows simulated values when the considered design included one site per reef location. As can be seen, the approximation appears to be reasonable for the next sampling

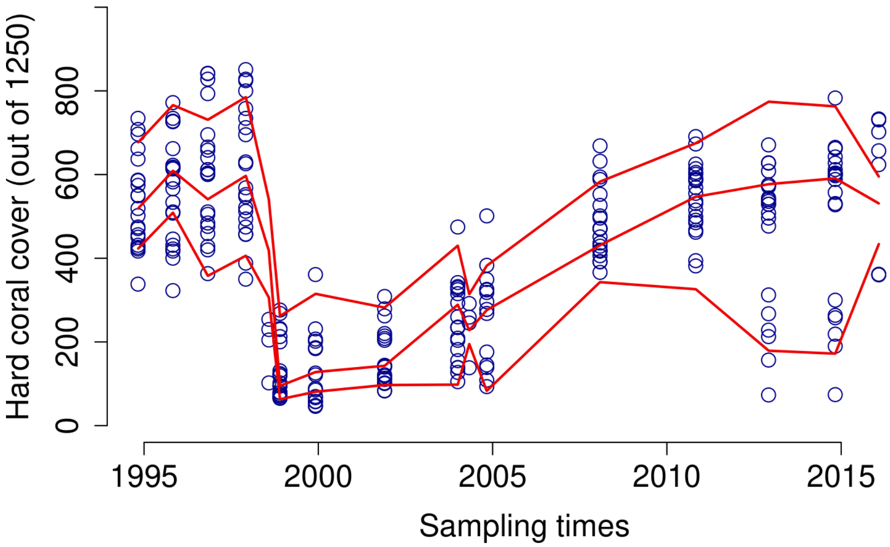

Figure A.3: Overall posterior predictive pattern based on the final model choice. The red lines represent the median and the lower and upper bounds of the 95% credible intervals for the prediction.

point (i.e. 2016.33). Given this, we propose the approach can be used to simulate supposed future data (line 4 in Algorithm 1) when determining adaptive designs.

A.8. Disturbance scenarios

Representatives from AIMS provided us with two disturbance scenarios (i.e. two values for $\boldsymbol{z}$) for which we evaluated designs (Table A.7). In Table A.7, the first row includes the covariates at the last survey time used for modelling where the covariate values represent the existing disturbance conditions between 2014.83 and 2016.08. The changes to the disturbance conditions when moving from the last survey time considered in this study (i.e. 2016.08) to the next survey time (i.e. 2016.33) under disturbance Sce-

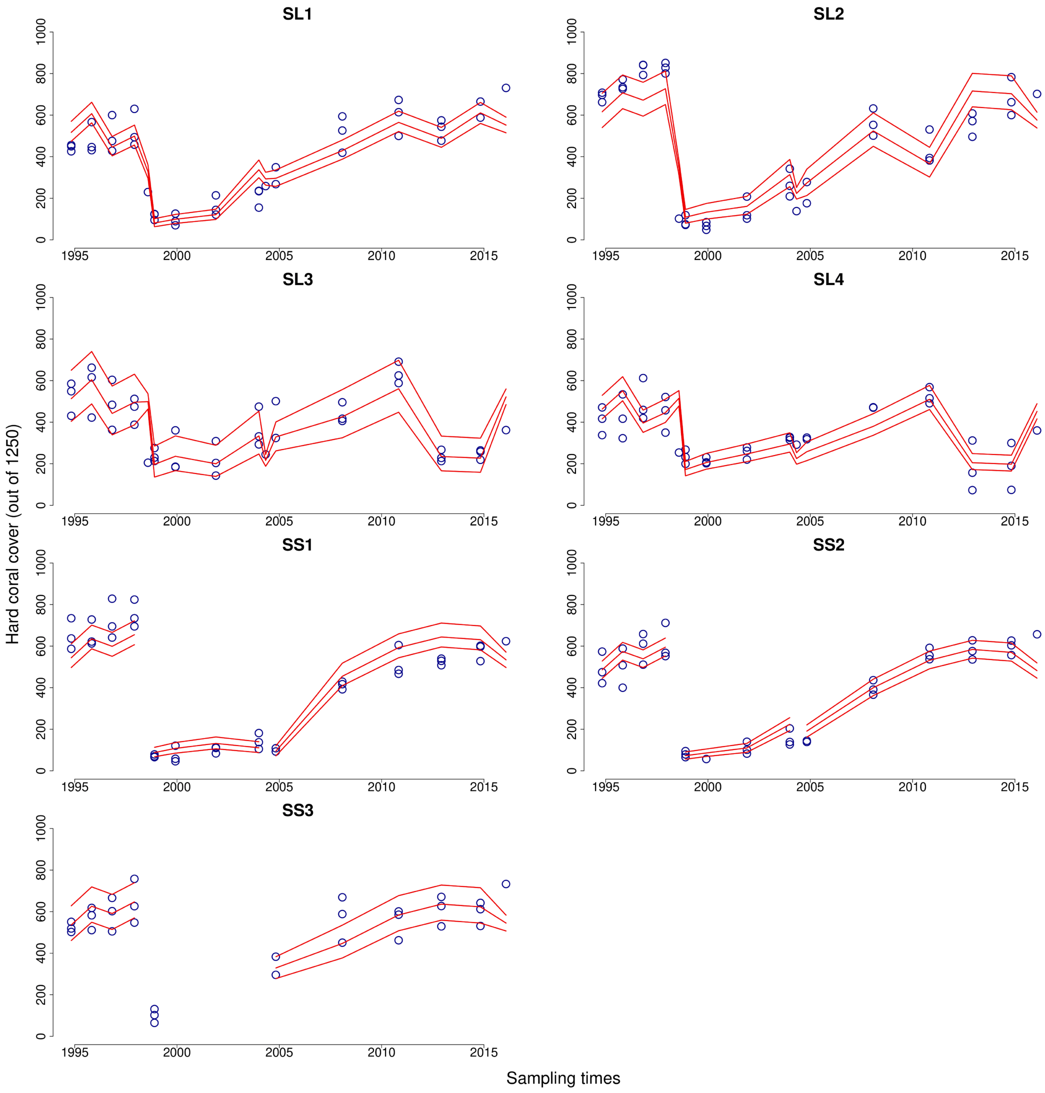

Figure A.4: Posterior predictive patterns based on the final model choice at each reef location. The red lines represent the median and the lower and upper bounds of the 95% credible intervals for the prediction.

narios (a) and (b) are provided in the second and third rows, respectively.

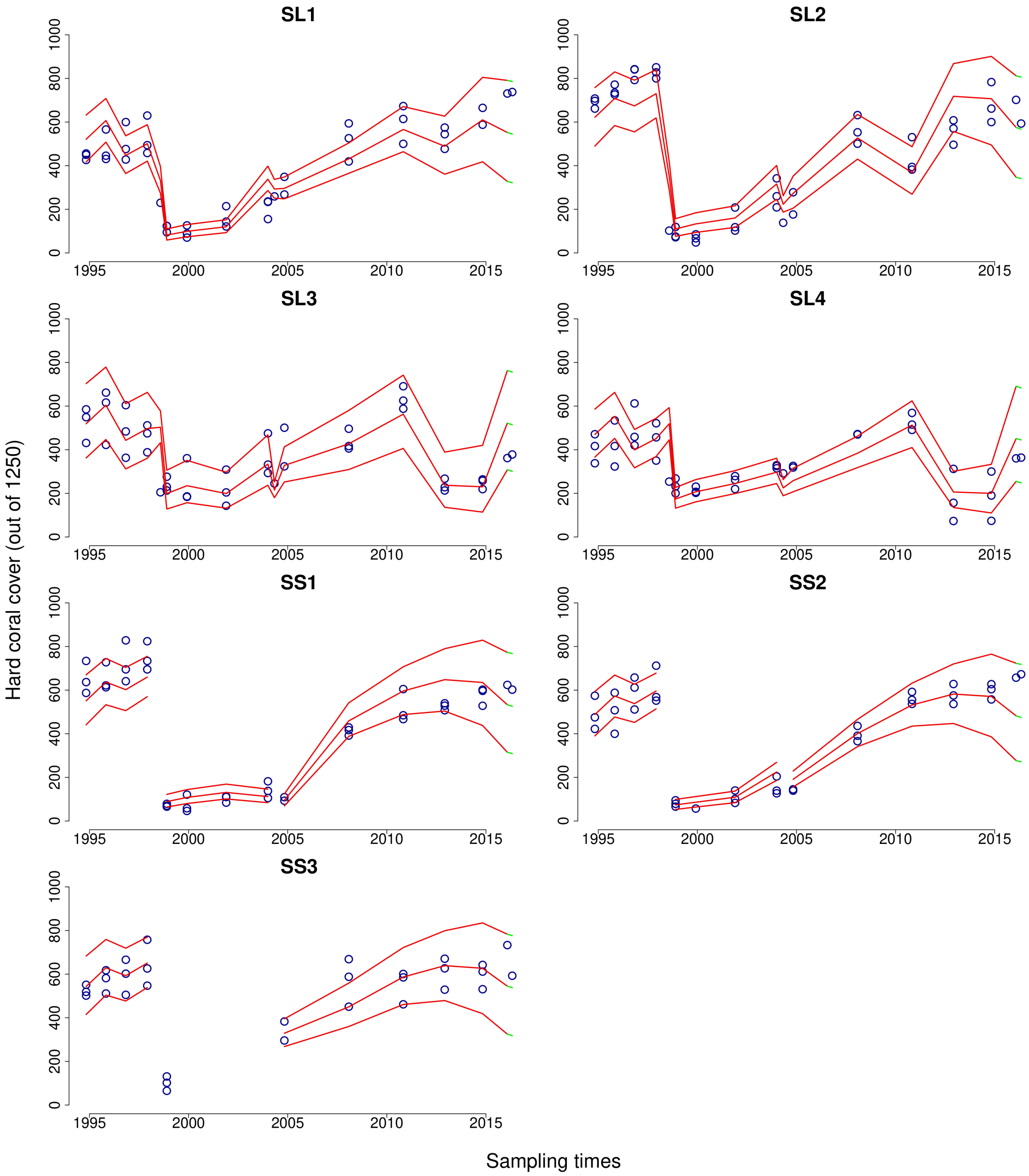

Figure A.5: Extrapolation of mean curves by reef locations to the next survey time. The red curves represent the median (i.e. median of mean values) and the corresponding lower and upper bounds of the 95% credible intervals for the “seen” data. Extrapolated curves up to the next survey time are shown in green colour.

Table A.7: Covariate values at the last survey time considered for modeling (i.e. 2016.08) and under two disturbance scenarios adopted in this study. Here, covariates at a given sampling time point have been defined based on what happened in between sampling times. Accordingly, the changes occurred in the covariates under each scenario compared with the last survey time are highlighted in red colour.

Survey times/ Scenarios	Reef location	Bleaching	Cyclone hours	Cyclone Loc	Severe cyclone Loc	Bleaching Loc	Bleaching × Cyclone
The last survey	SL1	1	6.928	0	0	1	0
time used for	SL2	1	6.928	0	0	1	0
modelling	SL3	1	6.928	0	0	1	0
historical data	SL4	1	6.928	0	0	1	0
(i.e. 2016.08)	SS1	1	6.928	0	0	1	0
	SS2	1	6.928	0	0	1	0
	SS3	1	6.928	0	0	1	0
Scenario (a)	SL1	1	0	0	0	1	0
New disturbances	SL2	1	0	0	0	1	0
at time 2016.33	SL3	1	0	0	0	1	0
Changes occurred:	SL4	1	0	0	0	1	0
Removed cyclone	SS1	1	0	0	0	1	0
impacts	SS2	1	0	0	0	1	0
	SS3	1	0	0	0	1	0
Scenario (b)	SL1	1	6.928	1	0	1	1
New disturbances	SL2	1	6.928	1	0	1	1
at time 2016.33	SL3	1	6.928	1	0	1	1
Changes occurred:	SL4	1	6.928	1	0	1	1
Added cyclone	SS1	1	6.928	1	0	1	1
location impacts	SS2	1	6.928	1	0	1	1
and an interaction	SS3	1	6.928	1	0	1	1

A.9. Utility evaluations

In the case where the prior and the posterior distributions both are Multivariate Normal, then the KLD utility function can be defined as follows:

$$u(\boldsymbol{d}, \boldsymbol{y}|\boldsymbol{d}_h, \boldsymbol{y}_h) = \frac{1}{2}\Bigg(\mathrm{tr}((\boldsymbol{\Sigma}^*_{m_1})^{-1}\boldsymbol{\Sigma}^*_{m_0}) + (\boldsymbol{\theta}^*_{m_1} - \boldsymbol{\theta}^*_{m_0})^T \times (\boldsymbol{\Sigma}^*_{m_1})^{-1} \times (\boldsymbol{\theta}^*_{m_1} - \boldsymbol{\theta}^*_{m_0}) - q_m + \ln\left(\frac{\det\boldsymbol{\Sigma}^*_{m_1}}{\det\boldsymbol{\Sigma}^*_{m_0}}\right)\Bigg), \tag{A.1}$$

where $\boldsymbol{\theta}^*_{m_0}$, $\boldsymbol{\theta}^*_{m_1}$ and $\boldsymbol{\Sigma}^*_{m_0}$, $\boldsymbol{\Sigma}^*_{m_1}$ are the means and variance covariance matrices of the prior and posterior distributions, respectively and q_m is the dimension of the parameter space.

A.10. Informative sites at each reef location

We determine the optimal design that comprises of the most informative site at each reef location subject to two disturbance scenarios under Objective (ii). The selected sites from each reef location into the optimal designs and expected utility values under the two scenarios are provided in Table A.8.

Table A.8: The optimal designs selected sites and expected utility values under Scenario (a) and (b) for Objective (ii). The red and green colours represent sites selected into optimal designs under Scenario (a) and (b), respectively.

Reef locations	Scenario (a)		Scenario (b)	
	Optimal design	Expected utility	Optimal design	Expected utility
	1	50.05	1	66.16
SL1	2	49.63	2	63.82
	3	49.46	3	64.86
	4	50.05	4	66.16
SL2	5	49.70	5	65.77
	6	49.55	6	64.77
	7	50.05	7	66.16
SL3	8	49.36	8	64.82
	9	49.09	9	64.66
	10	50.05	10	66.16
SL4	11	49.45	11	65.71
	12	49.29	12	65.47
	13	50.05	13	66.16
SS1	14	49.76	14	65.02
	15	50.03	15	64.51
	16	50.05	16	66.16
SS2	17	49.81	17	64.22
	18	49.48	18	63.88
	19	50.05	19	66.16
SS3	20	49.41	20	65.64
	21	49.38	21	66.11
